# Sex-specific risk factors for early mortality and survival after surgery of acute aortic dissection type a: a retrospective observational study

**DOI:** 10.1186/s13019-020-01189-w

**Published:** 2020-06-18

**Authors:** Christine Friedrich, Mohamed Ahmed Salem, Thomas Puehler, Grischa Hoffmann, Georg Lutter, Jochen Cremer, Assad Haneya

**Affiliations:** Department of Cardiovascular Surgery, University Hospital of Schleswig-Holstein, Campus Kiel, Arnold-Heller-Str. 3, Hs. C, 24105 Kiel, Germany

**Keywords:** Aorta, Dissection, Gender, Risk factors, Survival

## Abstract

**Background:**

Although gender-related disparities in cardiovascular surgery have been investigated extensively in the past decades, knowledge about the impact of gender on outcomes after surgery for acute aortic dissection type A (AADA) is sparse. This study investigated the impact of gender on early morbidity and mortality and follow-up outcome in patients after surgery for AADA and to analyze gender-related risk factors for 30-day mortality.

**Methods:**

This retrospective study included 368 consecutive patients (male 65.8% vs. female 34.2%) undergoing surgery for AADA between 2001 and 2016 at our department. Survival was estimated by Kaplan-Meier curves. Risk factors for 30-day mortality were assessed by multivariable logistic regression and interaction analysis.

**Results:**

Women were older (70.7 years vs. 60.6 years; *p* <  0.001) and showed a higher logistic EuroSCORE I (31.0% vs. 19.7%, p <  0.001). In the male group, a higher portion of smokers (27.6% vs. 16.0%, *p* = 0.015) and intraoperatively, more complex procedures and longer cardiopulmonary bypass (CPB) (171 min vs. 149 min, *p* = 0.001) and cross-clamping times (94 min vs. 85 min, *p* = 0.018) occurred. 30-day mortality was 19.0% in the female and 16.5% in the male group (*p* = 0.545). Predictive for 30-day mortality in both genders was intraoperative blood transfusion, while in the female group chronic obstructive pulmonary disease (COPD), peripheral arterial disease and preoperative intubation were predictive. Preoperative cardiopulmonary resuscitation and duration of CPB time were predictors only in males. Averaged follow-up time was 5.2 years and survival did not differ between genders, even if it was stratified by age over 70 years.

**Conclusions:**

This analysis demonstrated a similar and satisfactory survival in both genders after surgical treatment of AADA. Women and men differed significantly in age, unadjusted and adjusted risk factors and complexity of surgical treatment, but gender itself was no risk factor for mortality. These results suggest that the decision-making for surgical treatment should not depend on gender, but that accounting for sex-specific risk factors rather than common risk factors may help to improve the outcome in both genders.

## Background

Acute aortic dissection type A (AADA) is one of the major causes of death in cardiovascular disease for women and men [1, 2] and requires early surgical treatment to avoid live-threatening complications like aortic rupture, tamponade and malperfusion. Men are affected by AADA about twice as often compared to the incidence in women and present at younger age [2–4] and with considerable differences in comorbidities and risk factors [5–7]. The impact of gender in cardiovascular surgery has been investigated extensively during the past decades and women were shown to have worse outcomes after coronary bypass surgery [8, 9]. Due to advanced diagnostic methods, increased use of arterial grafts and surgical improvements, this gender gap seems to be slowly decreasing [9, 10].

Studies on gender-related differences after surgical repair of AADA are rarer and outcomes are controversial. A higher morbidity and mortality in women compared to that of men was reported previously [2, 5, 11]. As in coronary disease, delayed presentation and diagnosis in women due to less typical pain, older age and different comorbidities were described as potential risk factors for a worse outcome in female patients after surgery for AADA [2, 5]. In contrast to these findings, female gender was not associated with a worse outcome in recent studies [3, 4, 12, 13]. Several studies on the risk factors for mortality after surgery for AADA included gender as risk factor [4, 14], but information about sex-specific risk factors of women and men is rare.

The aim of this large retrospective observational study was to investigate the differences between women and men operated on for AADA regarding clinical presentation, management, surgical technique and outcomes in terms of morbidity, complications and survival. Gender-specific risk factors for 30-day mortality were detected based on a comprehensive database.

## Methods

### Study design and patients

From January 2001 to November 2016, a total of 368 consecutive patients suffering from AADA, 126 women and 242 men underwent surgical treatment of AADA at our department. AADA was defined as any dissection of the aortic wall with a clear entry site or intramural hematoma involving the ascending aorta or with an entry in the descending aorta with retrograde dissection. The diagnostic tool in all cases was computed tomography; echocardiography was used as confirmation when possible. Angiography and magnetic resonance imaging were not performed routinely. Primary endpoint was 30-day-mortality; secondary endpoints were peri- and postoperative complications and follow-up survival.

### Pre-, perioperative and postoperative data

Pre, intra- and postoperative variables were extracted retrospectively from electronic patient charts. They included demographics, comorbidities, history, clinical presentation, imaging findings, management, peri- and postoperative events and mortality. Predicted 30-day-mortality was calculated by logistic EuroSCORE I and EuroSCORE II (European System for Cardiac Operative Risk Evaluation) [15, 16]. Follow-up was conducted in September 2018 and long-term survival was evaluated by information given by the registry office.

### Operative technique and postoperative treatment

A standard median sternotomy followed by longitudinal pericardiotomy was performed under general anesthesia. All operations were performed by senior surgeons. The cardiopulmonary bypass (CPB) was performed with moderate hypothermic cardiac arrest (MHCA) with a nasopharyngeal temperature between 20 and 24 °C. In the early years either echo guided direct cannulation of the distal ascending aorta or, after surgical cut-down, the femoral artery was used for arterial cannulation in the most of cases. Since 2010, a transatrial cannulation of the left ventricle via the right upper pulmonary vein was used as standard technique for arterial cannulation [17]. Venous drainage was performed either through cannulation of the femoral vein or the right atrium with common two-stage venous cannula. A standard retrograde injection of cold blood cardioplegic solution for myocardial protection was performed in all cases. In complex cases requiring prolonged MHCA, a bilateral antegrade cerebral perfusion with oxygenated cold blood (18 °C) was introduced through a balloon catheter inserted in arch vessels with a pressure control of 50–60 mmHg. After suturing of the distal anastomosis, residual air was removed by restarting retrograde perfusion via the venous cannula followed by slow antegrade perfusion. Continuous CO_2_ insufflation was used in every case. After insertion of the perfusion cannula directly in the graft CPB restarted again. The proximal anastomosis and any required extra procedures (coronary artery bypass grafting (CABG), valve, etc.) were performed during re-warming.

### Statistics

Characteristics of women and men were compared by univariate statistics. Normally distributed continuous variables were presented as mean ± standard deviation and compared by unpaired *t*-test. Not normally distributed data as well as ordinal data were shown as median and the 25th and 75th percentiles or, where appropriate, as range and compared by Mann–Whitney *U*-test. Categorical data were summarized as absolute (n) and relative (%) frequencies and compared by Chi^2^-test or Fisher’s exact test. Missing data were excluded pairwise. Variables with missing data > 5% are marked in the results tables.

Gender, pre- and intraoperative variables were assessed for association to 30-day mortality by univariate analyses. Variables with a *p*-value of 0.1 or less (Table 4) were selected due to clinical relevance and included into multivariable logistic regression analysis with backward elimination to determine their relative impact (adjusted odds ratio, OR) on 30-day mortality. In the female group age, preoperative intubation, cardiopulmonary resuscitation (CPR), chronic obstructive pulmonary disease (COPD), peripheral arterial disease (PAD), coronary heart disease, hyperlipoproteinemia, chronic renal insufficiency, CPB time, number of red blood cell concentrate (RBC) and additional CABG were selected, while in the male group age, preoperative intubation, cardiogenic shock, CPR, CPB time, number of RBC and additional CABG were selected for multiple logistic regression analysis with a goodness of fit, described by Cox-Snell-R-Squared, of 0.199 and 0.178 in the female and male group, respectively. A second multivariable analysis including only the preoperative factors was carried out with a goodness of fit, described by Cox-Snell-R-Squared, of 0.217 and 0.118 in the female and male group, respectively.

In addition, interactions between sex and prognostic factors were analyzed by logistic regression analysis to detect sex-specific risk factors for 30-day mortality.

Survival of women and men was estimated on right-censored data by Kaplan-Meier curves for the total study population, for younger patients (< 70 years) and elderly patients (≥ 70 years) and was compared regarding gender-specific differences by log-rank test.

All tests were conducted 2-sided and a *p*-value of ≤0.05 was considered statistically significant. Data were analyzed with IBM SPSS Statistics for Windows (Version 24.0).

## Results

### Preoperative characteristics

One hundred twenty-six female patients (34.2%) and 242 male patients (65.8%) were included in the present study (Table 1). Women were about 10 years older than men (70.7 years vs. 60.6 years) at time of surgery and showed a significantly higher logistic EuroSCORE I (31.0% vs. 19.7%). Women had a smaller body surface area (1.8 m^2^ vs. 2.1 m^2^) and a lower body mass index (BMI 25.7 kg/m^2^ vs. 26.8 kg/m^2^) compared to men, but the percentage of obese (BMI > 30 kg/m^2^) patients was similar (18.4% vs. 21.1%). Men were more often smokers than women (27.6% vs. 16.0%). Male patients had significantly higher creatinine levels than female patients (98.1 μmol/l vs. 80.0 μmol/l), but the percentage of patients diagnosed with renal insufficiency or failure did not differ statistically between genders. According to the classification by DeBakey, women and men tended to present with a different extent of aortic dissection, however this difference was not statistically significant (*p* = 0.053). There were no further significant differences with regard to clinical presentations between genders.

**Table 1:** Demographic and clinical characteristics of the study population

Variable	All Patients (*n* = 368)	Male (*n* = 242)	Female (*n* = 126)	*p*- value
Age, years	62.8 ± 12.4 63.7 (54.2;72.5)	60.4 ± 12.0 60.6 (52.2;69.1)	67.5 ± 11.8 70.7 (60.3;76.4)	**< 0.001**
Logistic EuroSCORE I	23.1 (12.2;40.5)	19.7 (10.2;35.3)	31.0 (21.0;51.8)	**< 0.001**
EuroSCORE II	5.45 (3.06;12.41)	4.72 (2.75;11.63)	6.6 (4.0;13.4)	**0.005**
BSA (m^2^)	2.0 ± 0.2	2.1 ± 0.2	1.8 ± 0.2	**< 0.001**
Body mass index (kg/m^2^)	26.3 (23.9;29.0)	26.8 (24.5;29.3)	25.7 (23.1;28.1)	**0.005**
Body mass index > 30 (kg/m^2^)	74 (20.2%)	51 (21.1%)	23 (18.4%)	0.545
*Comorbidities*				
Arterial hypertension	262 (73.0%)	170 (72.6%)	92 (73.6%)	0.847
Pulmonary hypertension	4 (1.1%)	2 (0.9%)	2 (1.6%)	0.613
Type 2 Diabetes mellitus	21 (6.0%)	10 (4.4%)	11 (8.9%)	0.090
- Insulin dependent	7 (2.0%)	4 (1.8%)	3 (2.4%)	0.701
Hyperlipoproteinemia	50 (14.3%)	34 (15.0%)	16 (13.0%)	0.604
COPD	22 (6.2%)	12 (5.2%)	10 (8.0%)	0.289
Peripheral arterial disease	16 (4.5%)	9 (3.9%)	7 (5.6%)	0.454
Current Smoking*	71 (23.4%)	56 (27.6%)	15 (16.0%)	**0.015**
Atrial fibrillation	51 (13.9%)	32 (13.3%)	19 (15.1%)	0.636
Marfan syndrome	9 (2.5%)	7 (3.0%)	2 (1.6%)	0.504
Bicuspid aortic valve	9 (2.4%)	8 (3.3%)	1 (0.8%)	0.174
Neurological deficits	73 (20.3%)	45 (19.3%)	28 (22.2%)	0.513
*Preoperative state and cardiovascular risk profile*				
DeBakey type I**	259 (78.5%)	181 (81.5%)	78 (72.2%)	0.053
DeBakey type II**	71 (21.5%)	41 (18.5%)	30 (27.8%)	0.053
Creatinine (μmol/l)***	88.8 (72.2;114.0)	98.1 (80.0;120.5)	80.0 (63.8;95.3)	**< 0.001**
Chronic renal insufficiency	48 (13.6%)	36 (15.7%)	12 (9.8%)	0.120
Renal replacement therapy	7 (2.0%)	4 (1.7%)	3 (2.4%)	0.700
Coronary heart disease	63 (18.0%)	39 (17.3%)	24 (19.4%)	0.625
Previous PCI	24 (6.8%)	15 (6.5%)	9 (7.2%)	0.808
Previous cardiac surgery	12 (3.4%)	9 (3.9%)	3 (2.4%)	0.552
IABP/ECLS	5 (1.4%)	5 (2.1%)	0 (0.0%)	0.170
Aortic valve stenosis****	10 (2.9%)	4 (1.8%)	6 (5.1%)	0.097
Aortic valve insufficiency****	137 (40.1%)	86 (38.2%)	51 (43.6%)	0.337
Acute myocardial infarction	14 (3.8%)	8 (3.4%)	6 (4.8%)	0.570
Cardiogenic shock	27 (7.4%)	17 (7.2%)	10 (7.9%)	0.792
CPR (≤ 48 h)	31 (8.5%)	20 (8.4%)	11 (8.7%)	0.915
Intubated at admission	39 (10.7%)	25 (10.5%)	14 (11.2%)	0.828
*Diagnostic imaging (additional to CT)*				
Coronary angiography	130 (36.5%)	81 (34.9%)	49 (39.5%)	0.390
Magnetic resonance Imaging	9 (2.5%)	6 (2.6%)	3 (2.4%)	1.000

*BSA* Body surface area, *COPD* Chronic obstructive pulmonary disease, *PCI* Percutaneous coronary intervention, *IABP* Intra-aortic balloon pump, *ECLS* Extracorporeal life support, *CPR* Cardiopulmonary resuscitation; *18% missing values, **10% missing values; ***19% missing values, ****7% missing values; bold text, *p*-values ≤0.05; italic text, subheadings

### Perioperative findings and complications

Intraoperatively, males underwent more complex procedures when operated on for AADA and accordingly longer procedural times were found compared to those in the female group. Women received isolated supracoronary ascending aortic replacement (61.9% vs. 44.6%) more often while men received more David operations (8.3% vs. 1.6%) and other complex procedures, but the latter without reaching statistical significance (Table 2). In consequence, length of surgery, cardiopulmonary bypass time, cross-clamping times and circulatory arrest times were significantly longer in the male group. Additional aortic valve replacement was slightly more frequent in men (17.4% vs. 11.9%, *p* = 0.164) with a larger size of valve prostheses compared to that in women (25 mm vs. 23 mm). Women received more packed red blood cells (4 units vs. 2 units) and hemofiltration (34% vs. 23%) compared to men.

**Table 2:** Operative data

Variable	All Patients (*n* = 368)	Male (*n* = 242)	Female (*n* = 126)	*p*- value
Length of surgery (min)	280 (227;345)	287 (235;356)	254 (214;317)	**0.001**
Cardiopulmonary bypass time (min)	166 (135;212)	171 (139;220)	149 (124;196)	**0.001**
Cross-clamp time (min)	89 (70;127)	94 (72; 135)	85 (64;114)	**0.018**
Circulatory arrest (min)	33 (26;46)	35 (26;51)	30 (25;40)	**0.009**
Number of packed red blood cells (unit) *	4 (0–20)	2 (0–20)	4 (0–20)	**< 0.001**
Number of fresh frozen plasma (unit)*	0 (0–21)	0 (0–21)	2 (0–17)	0.500
Number of platelets (unit)*	2 (0–5)	2 (0–5)	2 (0–5)	0.168
*Surgical procedure*				
Isolated supracoronary replacement	186 (50.5%)	108 (44.6%)	78 (61.9%)	**0.002**
Partial arch replacement	78 (21.2%)	55 (22.7%)	23 (18.3%)	0.319
Total arch replacement	48 (13.0%)	37 (15.3%)	11 (8.7%)	0.076
- Elephant-trunk	7 (1.9%)	7 (2.9%)	0 (0.0%)	0.101
Conduit / Bentall operation	63 (17.1%)	44 (18.2%)	19 (15.1%)	0.453
David operation	22 (6.0%)	20 (8.3%)	2 (1.6%)	**0.010**
Additional AVR	57 (15.5%)	42 (17.4%)	15 (11.9%)	0.165
Additional CABG	35 (9.6%)	19 (8.0%)	16 (12.7%)	0.147
Diameter of ascending aorta prostheses (mm)	28 (28;30)	28 (28;30)	28 (26;30)	0.677
Additional MVR	1 (0.3%)	1 (0.4%)	0 (0.0%)	1.000
TEVAR/EVAR	23 (6.3%)	19 (7.9%)	4 (3.2%)	0.077
*Arterial cannulation*				
Femoral artery	78 (21.2%)	46 (19.0%)	32 (25.4%)	0.155
Ascending aorta	102 (27.7%)	67 (27.7%)	35 (27.8%)	0.985
Aortic arch	14 (3.8%)	9 (3.7%)	5 (4.0%)	1.000
Subclavian artery	1 (0.3%)	1 (0.4%)	0 (0.0%)	1.000
Apex	5 (1.4%)	4 (1.7%)	1 (0.8%)	0.664
Pulmonary vein	168 (45.7%)	115 (47.5%)	53 (42.1%)	0.319
Venous cannulation**				
Right atrium	330 (59.9%)	212 (95.1%)	118 (97.5%)	0.394
Bicaval	4 (1.2%)	4 (1.8%)	0 (0.0%)	0.302
Femoral vein	10 (2.9%)	7 (3.1%)	3 (2.5%)	1.000
Hemofiltration ***	83 (26.8%)	47 (23.0%)	36 (34.0%)	**0.039**

*AVR* Aortic valve replacement, *MVR* Mitral valve reconstruction / replacement, *CABG* Coronary artery bypass graft, *TEVAR* Thoracic endovascular aortic repair, *EVAR* Endovascular aortic repair; * 9–10% missing values, **6.5% missing values ***16% missing values; bold text, *p*-values ≤0.05; italic text, subheadings

### Postoperative data and outcome

Postoperative complications were more frequent in the male group. Blood drainage loss within 48 h postoperative was higher in men and likewise the total number of red blood cells, fresh frozen plasma and of platelets (see Table 3). Reintubation was necessary more often in the male group compared to the female group (21.2% vs. 11.9%) as well as tracheostomy due to long-term ventilation (26.1% vs. 16.7%). Moreover, the incidence of postoperative delirium (23.8% vs. 9.5%) and bronchopulmonary infection (15.8% vs. 7.9%) was higher in the male group. Women and men showed a similar incidence of acute kidney injury (AKI) according to the KDIGO (Kidney Disease: Improving Global Outcomes) guidelines and frequency of new-onset hemodialysis, but the duration of hemodialysis was longer in men (6 days vs. 3 days). No significant differences were noted between genders with regard to TIA or stroke, myocardial infarction and 30-day mortality (19.0% vs. 16.5%).

**Table 3:** Postoperative data and outcomes

Variable	All Patients (*n* = 368)	Male (*n* = 242)	Female (*n* = 126)	*p*-value
48 h-drainage loss (ml)*	850 (500;1300)	885 (500;1458)	750 (350;1150)	**0.034**
Postoperative blood transfusion	247 (70.8%)	166 (72.2%)	81 (68.1%)	0.424
Total number of packed red blood cells, unit	3 (0–56)	3.5 (0–56)	2 (0–21)	**0.030**
Total number of fresh frozen plasma, unit	0 (0–76)	0 (0–76)	0 (0–25)	**0.027**
Total number of platelets, unit	0 (0–20)	0 (0–20)	0 (0–7)	**0.002**
IABP/ECLS	10 (2.8%)	8 (3.4%)	2 (1.7%)	0.504
Reintubation	66 (18.0%)	51 (21.2%)	15 (11.9%)	**0.028**
Tracheotomy	84 (22.9%)	63 (26.1%)	21 (16.7%)	**0.040**
Postoperative delirium	69 (18.9%)	57 (23.8%)	12 (9.5%)	**0.001**
Postoperative myocardial infarction	4 (1.1%)	3 (1.3%)	1 (0.8%)	1.000
TIA / Stroke	56 (15.3%)	40 (16.7%)	16 (12.7%)	0.316
Electrical cardioversion	26 (7.1%)	14 (5.8%)	12 (9.5%)	0.192
CPR	28 (7.7%)	16 (6.7%)	12 (9.5%)	0.334
Bronchopulmonary infection	48 (13.1%)	38 (15.8%)	10 (7.9%)	**0.033**
Bacteriaemia/sepsis	17 (4.6%)	10 (4.2%)	7 (5.6%)	0.549
Rethoracotomy	62 (16.9%)	45 (18.7%)	17 (13.5%)	0.209
Sternal wound infection/VAC revision	6 (1.6%)	6 (2.5%)	0 (0.0%)	0.097
AKI KDIGO	80 (22.0%)	52 (21.8%)	28 (22.6%)	0.858
New–onset of Hemodialysis	78 (21.4%)	51 (21.2%)	27 (21.8%)	0.892
Temporary dialysis (d)	4 (2;12)	6 (3;13.5)	3 (1;5)	**0.030**
Atrial fibrillation	40 (11.4%)	26 (11.1%)	14 (11.9%)	0.833
Ventilation time (h)	64 (21;188)	63 (21;237)	67 (19;145)	0.270
ICU time (d)	5 (2;11)	5 (2;12)	5 (2;9)	0.442
Postoperative days	11 (7;19)	11.5 (8;20)	9.5 (7;17)	0.090
Hospital Mortality	61 (17.0%)	38 (16.2%)	23 (18.4%)	0.604
Cardiac death	36 (56.3%)	22 (55.0%)	14 (58.3%)	**–**
Cerebral death	4 (6.3%)	2 (5.0%)	2 (8.3%)	**–**
Sepsis	3 (4.7%)	3 (7.5%)	0 (0.0%)	**–**
MOF	21 (32.8%)	13 (32.5%)	8 (33.3%)	**–**
30 d-mortality	64 (17.4%)	40 (16.5%)	24 (19.0%)	0.545

*IABP* Intra-aortic balloon pump, *ECLS* Extracorporeal life support, *TIA* Transient ischemic attack, *CPR* Cardiopulmonary resuscitation, *AKI* Acute kidney injury, *KDIGO* Kidney Disease: Improving Global Outcomes, *MOF* Multiple organ failure, * 8.2% missing values; bold text, *p*-values ≤0.05

### Follow up results

**Fig. 1:**
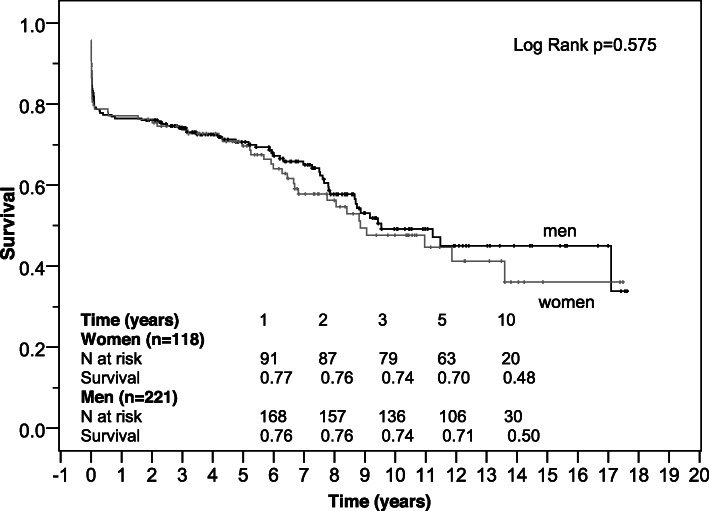
Kaplan-Meier survival curve of women and men after surgical repair of AADA

**Fig. 2:**
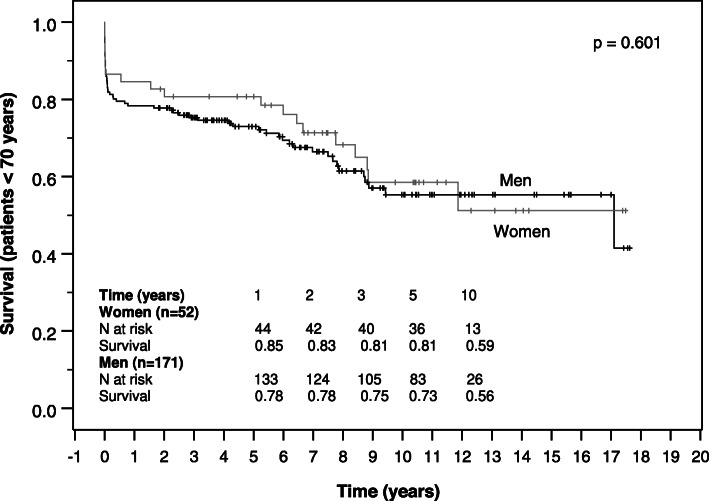
Kaplan-Meier survival curve of women and men aged younger than 70 years after surgical repair of AADA

**Fig. 3:**
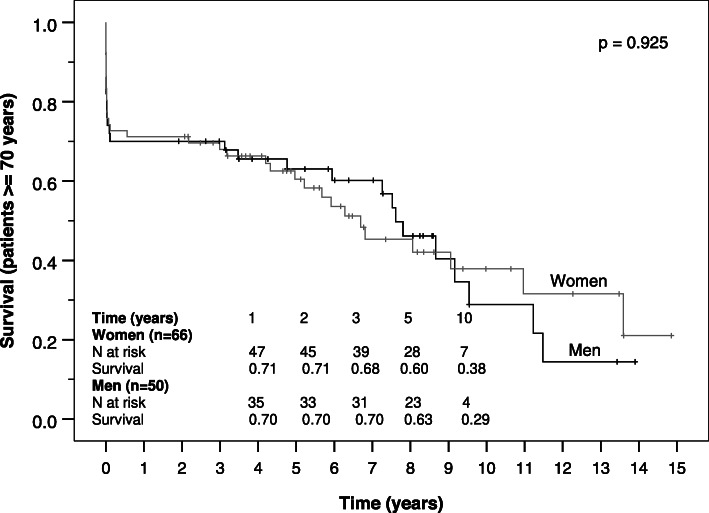
Kaplan-Meier survival curve of women and men aged 70 years or older after surgical repair of AADA

Follow up-completeness was 92.1% (*n* = 339). Median follow up time was 5.2 (1.9–8.4) years, and did not differ between genders (*p* = 0.542). Kaplan-Meier analyses showed no significant differences in all-cause survival between women and men (*p* = 0.575). Median estimated survival time was 8.9 (5.9–11.8) years in the female group and 9.5 (6.1–13.0) years in the male group. Cumulative survival of women and men was 77 and 76% after 1 year and 48 and 50% after 10 years (Fig. 1). Survival of women and men did not differ even if stratified by age. But as expected, it was lower in the elderly group aged 70 years and older with a median survival time of 6.5 and 7.8 years in female and male patients, respectively compared to 11 years in both genders younger than 70 years (Figs. 2 and 3).

### Risk factors for 30-day mortality

Crude predictors for 30-day-mortality differed substantially between the overall group, the female and the male group (Table 4). These findings were confirmed by interaction analysis for gender for the prognostic factors coronary heart disease, previous percutaneous coronary intervention (PCI), hyperlipoproteinemia and chronic renal insufficiency in the female group. However, COPD and PAD showed a tendency towards gender-specific association but failed significance. Cardiogenic shock was associated with 30-day mortality in the male group, but failed significance in the interaction analysis. Arterial hypertension, partial arch replacement and additional endovascular aortic repair were crude predictors only in male patients, but were inversely associated. The extent of aortic dissection according to the DeBakey type showed no significant association with 30-day mortality (both genders: *p* = 0.66, males: *p* = 0.27, females: *p* = 0.56). Detailed information on variables associated with 30-day mortality are provided as supplementary data.

**Table 4:** Pre- and intraoperative variables with univariate association to 30-d-mortality (*p*-values ≤0.10) and interaction between sex and risk factors (logistic regression analysis, *p*-values)

Variable	All Patients (*n* = 368)	Male (*n* = 242)	Female (*n* = 126)	Sex-specific Interaction
Total cases	368	242	126	**–**
Female gender	0.545			
Age, years	0.100	0.971	**0.026**	0.123
Logistic EuroSCORE I (%)	**< 0.001**	**0.003**	**< 0.00**1	0.599
Cardiopulmonary resuscitation 48 h preoperative	**< 0.001**	**< 0.001**	**< 0.001**	0.226
Cardiogenic shock		**0.037**		0.266
Intubated	**< 0.001**	**0.001**	**0.004**	0.821
Chronic obstructive pulmonary disease			**0.018**	0.059
*Arterial hypertension*	*0.010*	*0.004*		0.198
Peripheral arterial disease			**0.019**	0.083
Coronary heart disease	**< 0.001**		**< 0.001**	**0.009**
Previous percutaneous coronary intervention	**0.007**		**< 0.001**	**0.005**
Previous cardiac surgery	**0.005**		**0.006**	0.999
Diabetes mellitus type II	0.059			0.928
Hyperlipoproteinemia			**0.009**	**0.005**
Creatinine (μmol/l) preoperative^a^	**0.007**	**0.001**	0.051	0.334
Chronic renal insufficiency			**0.039**	**0.017**
*Ascending aorta prostheses (mm)*	*0.088*			0.434
Length of surgery (min)	**0.001**	**0.006**	0.060	0.231
Cardiopulmonary bypass time (min)	**< 0.001**	**0.001**	**0.034**	0.855
Surgical procedure				
Additional coronary artery bypass graft	**< 0.001**	**0.018**	**0.003**	0.508
*Partial arch replacement*	*0.061*	*0.036*		0.232
*TEVAR/EVAR*		*0.050*		0.998
Number of RBC, (U)	**< 0.001**	**0.008**	**0.001**	0.658
Number of platelets, (U)	**< 0.001**	**0.048**	**0.001**	0.198

*TEVAR/EVAR*, (thoracic) endovascular aortic repair, *RBC* Red blood cell concentrate (unit)

^a^19% missing values; italic text, inverse association; bold text, *p*-values ≤0.05

In the multivariable analysis on pre- and intraoperative variables adjusted overall predictors for 30-day mortality were CPB time, number of intraoperatively administered red blood cell units, additional CABG and particularly cardiopulmonary resuscitation within 48 h preoperative (Table 5). In the female group COPD, PAD and preoperative intubation were strong predictors for 30-day mortality, while preoperative cardiopulmonary resuscitation and cardiopulmonary bypass time were predictors only in the male group. In both genders intraoperatively administered red blood cells units were significant predictors for 30-day mortality.

**Table 5:** Allover and sex-specific adjusted pre- and intraoperative risk factors for 30-day-mortality (variables with significant or nearly significant *p*-values presented)

Variable	Total			Male			Female		
	*p*-value	OR	CI	p-value	OR	CI	p-value	OR	CI
Age (years)	0.058	1.038	0.999–1.079						
DM 2	0.052	3.368	0.988–11.479						
COPD							**0.014**	9.255	1.579–54.234
PAD							**0.043**	7.274	1.065–49.682
Intubated							**0.005**	7.499	1.840–30.565
CPR	**< 0.001**	12.861	4.627–35.746	**< 0.001**	13.379	4.051–44.190			
CPB	**0.009**	1.008	1.002–1.014	**0.002**	1.009	1.003–1.015			
RBC	**0.006**	1.130	1.036–1.233	**0.046**	1.110	1.002–1.229	**0.020**	1.187	1.028–1.371
CABG	**0.021**	3.350	1.201–9.346						

*OR* Odds Ratio, *CI* Confidence interval, *DM 2* Diabetes mellitus type 2, *COPD* Chronic obstructive pulmonary disease, *PAD* Peripheral arterial disease, *CPR* Cardiopulmonary resuscitation (≤ 48 h preoperative), *CPB* Cardiopulmonary bypass time (min), *RBC* Number of red blood cell concentrates (unit) intraoperative, *CABG* Additional CABG surgery; bold text, *p*-values ≤0.05

In accordance with the interaction analysis, the multivariable analysis, in which only preoperative variables were included (Table 6) revealed coronary heart disease and hyperlipoproteinemia as predictors in the female patient group. Age also revealed as risk factor for women in the preoperative model, but age showed no significant association with gender in the interaction analysis.

**Table 6:** Allover and sex-specific adjusted preoperative risk factors for 30-day-mortality (variables with significant or nearly significant *p*-values presented)

Variable	Total			Male			Female		
	*p*-value	OR	CI	p-value	OR	CI	*p*-value	OR	CI
Age (years)							**0.019**	1.082	1.013–1.155
DM 2	0.052	2.847	0.990–8.188						
Hyperlipoproteinemia							**0.009**	6.786	1.611–28.596
Coronary heart disease	**0.011**	2.568	1.246–5.292				**0.004**	5.834	1.779–19.135
CPR	< **0.001**	12.272	5.194–28.996	**< 0.001**	16.727	5.834–47.954	**0.024**	6.973	1.297–37.500

*OR* Odds Ratio, *CI* Confidence interval, *DM 2* Diabetes mellitus type 2, *CPR* Cardiopulmonary resuscitation (≤ 48 h preoperative); bold text, *p*-values ≤0.05

## Discussion

Our large study compared early and late outcomes between women and men following surgical repair of AADA and assessed gender specific risk factors for 30-day mortality. Our main findings are that women were about 10 years older at the time of surgery and that men underwent more complex and longer surgery times with concomitant more complicated postoperative course. Early and late mortality did not differ significantly, though women and men showed substantial differences regarding unadjusted and adjusted risk factors for 30-day mortality.

Nienaber et al. [5] presented a first systematic study of the International Registry of Acute Aortic dissection (IRAD) on gender-related differences in patients operated on for AADA and showed that women were considerably older and less frequently affected by AADA than men. AADA is predominantly a disease of the elderly and women are affected about 6 to 10 years later than men [3, 4, 7], which is confirmed by our findings. The protective effect of ovarian estrogen on cardiovascular disease in premenopausal women and its decrease in the postmenopausal period [18] may promote the later onset of AADA. Women develop hypertension, one of the major risk factors for AADA, about a decade later than men due to the protective effect of estrogens on blood pressure in premenopause [19, 20]. However, the specific pathophysiological differences underlying AADA in women and men remain to be clarified.

Several studies indicated a poor outcome after surgery for AADA in women [5, 11] and Nienaber et al. [5] found a higher in-hospital mortality in women (32% vs. 22%), more in-hospital complications and a worse surgical outcome despite similar time from symptom onset to diagnosis, surgical technique and hemodynamics. In contrast female gender was not associated with worse early and long-term outcomes in several recent studies [3, 4, 12], which was confirmed by our results.

Age is a risk factor for early mortality in AADA in several studies [14, 21], especially in octogenarians [22]. Age was associated to 30-day mortality in the female group in our univariate analysis and in the multivariable analysis of the preoperative variables. The results of our interaction analysis point towards age as a risk factor in female gender, but without reaching significance. However, the multivariable analysis including pre- and intraoperative variables revealed that other risk factors had a higher impact on mortality than age in women and men which is in line with the findings of Suzuki et al. [23]. Fukui et al. [4] found gender and age not to be associated with an elevated early mortality.

Men and women presented with only few differences in preoperative findings, despite for age and EuroSCORE. As described previously [11], smoking as a major common risk factor for aortic dissection [11, 24] had a higher prevalence in the male group compared to the prevalence in females. Men presented with a higher creatinine level and tended to have a higher rate of chronic renal insufficiency, however, this difference was not significant. Consistent with our findings Fukui et al. [4], Conway et al. [3] and Sabashnikov et al. [12] reported increased creatinine levels in men despite no substantial disparities in GFR and rate of chronic renal failure, concluding that creatinine is of lower clinical relevance [12]. Moreover, creatinine levels are physiologically higher in men, as indicated by sex-specific standard values.

In contrast to previous findings women did not present with a higher incidence of arterial hypertension in our study despite their higher age [3, 5, 11]. However, hypertension was associated with lower 30-day mortality in the univariate analysis in the overall group and the male group, a tendency which was already observed in other studies [4, 21]. Maybe known arterial hypertension as major risk factor for AADA [24] promotes early diagnosis of AADA, which is mandatory due to an excessive high mortality rate of 1 to 2% every hour after its onset [2]. Chua and coworker [25] investigated risk factors for missed diagnosis in 68 patients and found that 69.0% of patients diagnosed with AADA presented with hypertension, while among patients with missed diagnosis for AADA, only 46.2% presented with hypertension. In theory, a history of arterial hypertension may direct more quickly to the diagnosis of AADA and its live-saving treatment.

Though men were younger and did not present with a considerably worse preoperative state, postoperative complications were more frequent compared to those in women, presumably associated with the higher portion of complex surgery. The decision to perform less complex surgery might be related to the older age of female patients and the usually less elaborate surgical treatment that is recommended in elderly patients to shorten bypass-time [7, 26]. Accordingly, procedural times were significantly longer in the male group which is in line with the report of Fukui et al. [4]. Boening et al. [22] showed that a longer operating time was a risk factor for neurologic dysfunction after AADA. However, in our study male patients had similar rates of postoperative stroke or TIA.

Intraoperative blood transfusion was a risk factor for 30-day mortality in both genders, but despite shorter procedural times and less complex surgery, female patients received more red blood cell units intraoperatively compared to male patients. Though data is lacking in our analysis, many studies showed that women have lower preoperative hematocrit prior to cardiovascular surgery, a higher rate of red blood cell transfusion and greater hemodilution on cardiopulmonary bypass compared to men [27, 28]. In a large analysis on 13,739 patients undergoing cardiac surgery, Mehta et al. [27] suggest that women have a better tolerance to hemodilution and that specific thresholds for blood transfusions in women may reduce its harmful effects.

Despite the observed gender-specific differences, women and men had an acceptable 30-day mortality of 19.0% vs. 16.5% and a total 30-day mortality of 17.4%, overall comparable to the results of Boening et al. and Rylski et al. who reported a total 30-day mortality of 16.9% [22], 16.6% in the male group but a lower mortality of 16.3% in the female group [13] in their large analysis of the German registry for Acute Aortic Dissection type A (GERAADA).

Crude and adjusted risk factors for 30-day mortality showed major differences between genders**.** Solely in the female group, PAD, coronary heart disease, previous PCI, previous cardiac surgery, as well as COPD, hyperlipoproteinaemia and chronic renal insufficiency were identified as specific predictors, which are predominantly associated with known age-related comorbidities atherosclerosis and prior cardiac surgery [21]. Moreover, renal insufficiency before cardiac surgery was discussed to have a stronger impact on the mortality in women [6]. In contrast, only in male patients, cardiogenic shock was a crude predictor. Shock and prior cardiac surgery were described as risk factors for early mortality after surgery for AADA [21], however, these risk factors appeared distinctly gender-specific in our study.

In the female group COPD was identified as strong predictor for 30-day mortality. COPD was a risk factor for in-hospital mortality after aortic surgery in a large analysis from the Japan Adult Cardiovascular Database [29]. Although women in our study were less often smokers, there are hints that women may be more prone to COPD and possibly develop a higher degree of pulmonary hyperinflation and mortality compared to men [30]. In contrast Suzuki et al. found COPD to be a specific risk factor only in men, which may be caused by the higher portion of men with smoking history in their study [23]. However, besides smoking, COPD should be considered in gender-specific risk analysis.

Patients with coronary malperfusion who underwent additional CABG with surgery for AADA showed a higher unadjusted risk for 30-day mortality in all groups. Malperfusion of different organs affected by dissection of the aortic branch vessels is one of the main complications of AADA and is a known risk factor for early mortality after surgical repair of AADA [14]. However, after adjustment it was not a risk factor in the female or male group.

Though the clinical presentation of women and men was comparable, preoperative cardiopulmonary resuscitation as strong risk factor for a poor outcome [14] showed a significant impact only in men in the pre- and intraoperative model, which is in line with the findings of Suzuki et al. [23]. Except for additional CABG, surgical techniques showed no significant impact on the early mortality. Cardiopulmonary bypass time as potential indicator for complex surgery was a significant predictor for 30-day mortality solely in the overall group and the male group. However, we could not prove an interaction to gender, corresponding to Suzuki et al. 2018 [23] who found CPB time to be a risk factor for women and men.

We therefore conclude that in general it is important to include intraoperative factors in multivariable analysis in order to draw conclusions about all risk factors for early mortality. However, a detailed separate analysis of the preoperative risk profile may be useful to predict the mortality in patients prior to surgery for AADA.

Kaplan-Meier analyses showed no significant differences in survival between women and men in our study as in recent studies [3, 4, 12, 23]. Cumulative survival of women and men in our study was similar to the results of a matched cohort of 71 female and male patients in the study of Sabashnikov et al. [12] after 1 year. After 5 years it was lower in their matched cohort (62.9% vs. 62.8%), which may be due to the older age compared to that of our patients. Age-stratified survival showed no significant differences between genders as well, though the portion of women was higher in the elderly group. The comparison of survival rates to other studies is aggravated since we did not exclude in-hospital mortality from our Kaplan-Meier analysis.

**Fig. 4:**
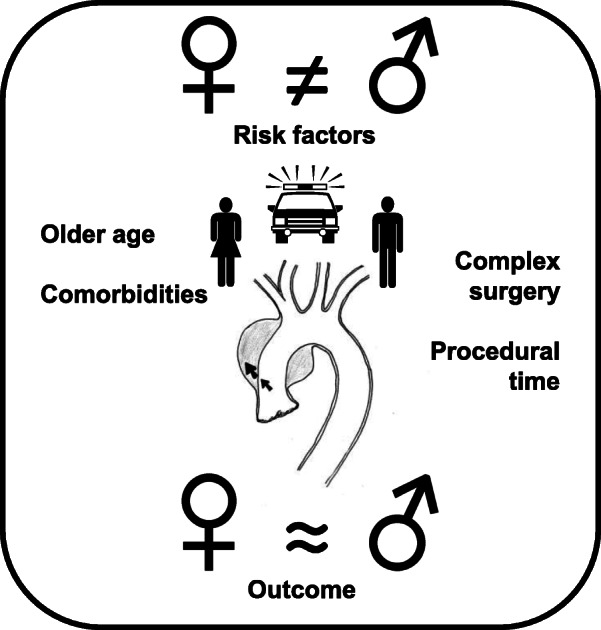
Visual representation of main findings

In summary, women and men showed a comparable short- and long-term survival after surgery for AADA, though women were significantly older and presented with a different pattern of risk factors and men underwent more complex surgery and had a more complicated postoperative course (Fig. 4). A similar incidence of hypertension in women and men in our study and in general, advances in surgical techniques and earlier diagnosis of AADA over time may have improved outcomes in female patients. Nevertheless, major differences in women and men regarding genetics, hormonal status and risk factors [6] need continued attention to improve outcomes in women and men alike.

### Study limitations

The principal limitation of this single-center study is its retrospective design and thus the issue of missing and incomplete data. The limited sample size in the female patient group and the large confidence intervals of some predictors should be considered. Studies on larger patient populations should further investigate sex-specific risk factors.

## Conclusions

A major finding of this study is that unadjusted and adjusted predictors for 30-day-mortality differed substantially between the overall patients, women and men. Survival was comparable and satisfactory in both genders and gender itself was no risk factor for mortality after surgical repair of AADA. Consideration of sex-specific risk factors instead of common risk factors may help to improve outcomes in women and men after surgical repair of AADA.

## Supplementary information

**Additional file 1: Supplementary Table 1.** Pre- and intraoperative variables with univariate association to 30-d-mortality (*p*-values ≤0.10), Supplement to Table 4 in original article.

## Data Availability

The datasets used and /or analysed during the current study are available from the corresponding author on reasonable request.

## Abbreviations

AADA: Acute aortic dissection type A
CPB: Cardiopulmonary bypass
COPD: Chronic obstructive pulmonary disease
EuroSCORE: European System for Cardiac Operative Risk Evaluation
MHCA: Moderate hypothermic cardiac arrest
CABG: Coronary artery bypass grafting
PAD: Peripheral arterial disease
OR: Odds ratio
RBC: Number of red blood cell concentrate
KDIGO: Kidney Disease: Improving Global Outcomes
PCI: Previous percutaneous coronary intervention
IRAD: International Registry of Acute Aortic dissection
GERAADA: German Registry for Acute Aortic Dissection type A
