# Supplementary material for: Sex-specific risk factors for early mortality and survival after surgery of acute aortic dissection type a: a retrospective observational study
Source: J Cardiothorac Surg. 2020 Jun 18;15:145. doi: 10.1186/s13019-020-01189-w (PMC7301454; doi:10.1186/s13019-020-01189-w)
Supplement: Supplementary file 1 — Additional file 1: Supplementary Table 1. Pre- and intraoperative variables with univariate association to 30-d-mortality (p-values ≤0.10), Supplement to Table 4 in original article. [file 13019_2020_1189_MOESM1_ESM.docx]

**Supplementary Table 1**: Pre- and intraoperative variables with univariate association to 30-d-mortality (p-values ≤ 0.10), Supplement to table 4 in original article

| **Variable** | **All Patients**  **Survivors**  (n = 304) | **Non-survivors**  (n = 64) | **p-value** | **Male**  **Survivors**  (n = 202) | **Male non-survivors**  (n = 40) | **p-value** | **Female survivors**  (n = 102) | **Female non-survivors**  (n = 24) |  | **p- value** |
| --- | --- | --- | --- | --- | --- | --- | --- | --- | --- | --- |
| Female gender | 33.6% | 37.5% | 0.545 |  |  |  |  |  |  |  |
| Age, years | 63 (55;71) | 68 (53;75) | 0.100 | 61 (53;68) | 58 (51;73) | 0.971 | 70 (59;76) | 73 (68;79) |  | **0.026** |
| Logistic ES I (%) | 21 (12;37) | 36 (22;57) | **<0.001** | 19 (10;32) | 29 (15;49) | **0.003** | 30 (14;47) | 51 (27;67) |  | **<0.001** |
| CPR 48 h preoperative | 3.7% | 31.7% | **<0.001** | 3.0% | 35.9% | **<0.001** | 4.9% | 25.0% |  | **<0.001** |
| Cardiogenic shock |  |  |  | 5.5% | 15.8% | **0.037** |  |  |  |  |
| Intubated | 7.0% | 28.6% | **<0.001** | 7.0% | 27.5% | **0.001** | 6.9% | 30.4% |  | **0.004** |
| Calcific aortic disease | 1.7% | 6.3% | 0.054 |  |  |  | 1.0% | 12.5% |  | **0.023** |
| COPD |  |  |  |  |  |  | 4.9% | 21.7% |  | **0.018** |
| Arterial hypertension | 75.7% | 59.3% | **0.010** | 76.3% | 52.8% | **0.004** |  |  |  |  |
| PVD |  |  |  |  |  |  | 2.9% | 18.2% |  | **0.019** |
| Coronary heart disease | 14.9% | 35.2% | **<0.001** |  |  |  | 11.8% | 54.5% |  | **<0.001** |
| Previous PCI | 5.0% | 15.8% | **0.007** |  |  |  | 2.0% | 30.4% |  | **<0.001** |
| Previous CABG | 2.0% | 10.5% | **0.005** |  |  |  | 0.0% | 13.0% |  | **0.006** |
| Diabetes mellitus type II | 4.7% | 12.3% | 0.059 |  |  |  |  |  |  |  |
| Hyperlipoproteinemia |  |  |  |  |  |  | 8.9% | 31.8% |  | **0.009** |
| Creatinine (µmol/l) preoperative* | 88 (71;107) | 102 (80;133) | **0.007** | 96 (79;115) | 121 (97;141) | **0.001** | 76 (62;89) | 87 (70;98) |  | 0.051 |
| Chronic renal insufficiency |  |  |  |  |  |  | 6.9% | 22.7% |  | **0.039** |
| Asc. aorta prosthesis (mm) | 28 (28;30) | 28 (26;30) | 0.088 |  |  |  |  |  |  |  |
| Length of surgery (min) | 274  (225;330) | 320 (240;415) | **0.001** | 281 (232;342) | 335 (264;460) | **0.006** | 253  (211;301) | 302 (225;354) |  | 0.060 |
| CPB time [min] | 161  (134;204) | 198 (148;247) | **<0.001** | 165 (139;211) | 212 (163;281) | **0.001** | 147  (121;192) | 187 (139;225) |  | **0.034** |
| **Surgical procedure** |  |  |  |  |  |  |  |  |  |  |
| Additional CABG | 6.6% | 24.2% | **<0.001** | 6.0% | 18.4% | **0.018** | 7.8% | 33.3% |  | **0.003** |
| Partial arch replacement | 23.0% | 12.5% | 0.061 | 25.2% | 10.0% | **0.036** |  |  |  |  |
| TEVAR/EVAR |  |  |  | 9.4% | 0.0% | **0.050** |  |  |  |  |
| Number of RBC, (U)** | 3 (0;6) | 5 (2;10) | **<0.001** | 2 (0;5) | 4 (0.3;10) | **0.008** | 4 (2;6) | 6 (5;9) |  | **0.001** |
| Number of platelets, (U)** | 2 (1;2) | 2 (2;3) | **<0.001** | 2 (1;2) | 2 (1;3) | **0.048** | 2 (1;2) | 2 (2;2.3) |  | **0.001** |

ES, EuroSCORE; CPR, Cardiopulmonary resuscitation; COPD, chronic obstructive pulmonary disease; PVD, peripheral vascular disease; PCI, percutaneous coronary intervention; CABG, coronary artery bypass graft; CPB, cardiopulmonary bypass; TEVAR, thoracic endovascular aortic repair; EVAR, endovascular aortic repair; RBC, red blood cell concentrate (unit); * 19% missing values, ** 9-10% missing values, bold text, p-values ≤ 0.05
